# Ischemic postconditioning attenuates liver warm ischemia-reperfusion injury through Akt-eNOS-NO-HIF pathway

**DOI:** 10.1186/1423-0127-18-79

**Published:** 2011-10-28

**Authors:** Jia Y Guo, Tong Yang, Xiang G Sun, Ni Y Zhou, Fu S Li, Dan Long, Tao Lin, Ping Y Li, Li Feng

**Affiliations:** 1 Key Laboratory of Transplant Engineering and Immunology of Health Ministry of China, West China Hospital, Sichuan University, Chengdu, 610041, Sichuan Province, P. R. China; 2 Transplantation Institute, Department of urology, West China Hospital, Sichuan University, Chengdu, 610041, Sichuan Province, P. R. China

## Abstract

**Background:**

Ischemic postconditioning (IPO) has been demonstrated to attenuate ischemia/reperfusion (I/R) injury in the heart and brain, its roles to liver remain to be defined. The study was undertaken to determine if IPO would attenuate liver warm I/R injury and its protective mechanism.

**Methods:**

Mice were divided into sham, I/R, IPO+I/R (occlusing the porta hepatis for 60 min, then treated for three cycles of 10 sec brief reperfusion consecutively, followed by a persistent reperfusion); L-NAME+ sham (L-NAME, 16 mg/kg, i.v., 5 min before repefusion); L-NAME+I/R; and L-NAME+ IPO. Blood flow of caudate and left lobe of the liver was blocked. Functional and morphologic changes of livers were evaluated. Contents of nitric oxide, eNOS and iNOS in serum were assayed. Concentration of eNOS, iNOS, malondialdehyde (MDA) and activity of superoxide dismutase (SOD) in hepatic tissue were also measured. Expressions of Akt, p-Akt and HIF-1α protein were determined by western blot. Expressions of TNF-α and ICAM-1 were measured by immunohistochemistry and RT-PCR.

**Results:**

IPO attenuated the dramatically functional and morphological injuries. The levels of ALT was significantly reduced in IPO+I/R group (p < 0.05). Contents of nitric oxide and eNOS in serum were increased in the IPO+I/R group (p < 0.05). IPO also up-regulated the concentration of eNOS, activity of SOD in hepatic tissue (p < 0.05), while reduced the concentration of MDA (p < 0.05). Moreover, protein expressions of HIF-1α and p-Akt were markedly enhanced in IPO+I/R group. Protein and mRNA expression of TNF-α and ICAM-1 were markedly suppressed by IPO (p < 0.05). These protective effects of IPO could be abolished by L-NAME.

**Conclusions:**

We found that IPO increased the content of NO and attenuated the overproduction of ROS and I/R-induced inflammation. Increased NO contents may contribute to increasing HIF-1α level, and HIF-1α and NO would simultaneously protect liver from I/R injury. These findings suggested IPO may have the therapeutic potential through Akt-eNOS-NO-HIF pathway for the better management of liver I/R injury.

## Background

Multiple studies have shown that ischemic preconditioning (IPC), defined as one or more brief ischemic insult, confers organ protection from I/R injury [1,2]. Although IPC has shown protective effects against I/R injury, its utilization as clinical strategy is largely limited because the onset of ischemia is difficult to be predicted. However, the onset of reperfusion is more predictable. Recently, a new strategy, named ischemic postconditioning (IPO), was described by Zhao et al [3] and showed promising results for cardiac reperfusion injury. It consists in application of several brief cycles of ischaemia and reperfusion, made soon after the ischemia phase and before reperfusion phase [3,4]. This method was used successfully in heart [5,6], brain [7], kidney [8,9], spinal cord [10], intestine [11] and, recently, a few study that demonstrate its efficacy in liver [12-14] I/R injury. Although the protective effects of IPO on several organs have been identified, the interventions among the multiple and interacting components involved in IPO remains unclearly understood. And so far, the exact protective mechanism of IPO on liver I/R injury have not been completely elucidated.

Several studies have suggested that NO protects organs against I/R injury [15,16]. The potentially protective role of endogenous NO in liver I/R injury is also supported by several studies. There is evidence implicating NO is involved in the heart [17] and kidney [18] protections of ischemic postconditioning, but there was no information as to whether NO participates in the protective response elicited by liver IPO.

Studies have shown that NO can upregulate the rate of hypoxia inducible factor-1α (HIF-1α) synthesis by activating the phosphatidylinositol 3-kinase (PI3K)-Akt [19,20] and blocks proline hydroxylase (PHD) activity [19]. Activation and upregulation of HIF-1α has been recently found to be able to protect liver from I/R [21,22]. Several studies also indicated that the PI3K/Akt pathway plays an important role in protective action of IPO [23,24], but mechanism by which PI3K/Akt pathway is involved in the liver IPO remain poorly understood. Furthermore, Akt is important in the activation of eNOS mediated NO production [25]. Studies have shown that cardioprotection is associated with NO production following Akt-mediated eNOS activation [26,27]. So we wonder if IPO treatment may have protective role against liver I/R injury through Akt-NO-HIF pathway. As such, the present study was undertaken to investigate the more detailed protective mechanism of IPO on liver I/R injury. Our data indicate that IPO may have the therapeutic potential through Akt-eNOS-NO-HIF pathway for the better management of liver I/R injury.

## Materials and methods

### N-nitro-L-arginine methylester (L-NAME)

N-nitro-L-arginine methylester (L-NAME), a non-selective nitric oxide synthase (NOS) inhibitor, were purchased from Sigma (St. Louis, MO, USA). In this study, L-NAME was dissolved and diluted with saline.

### Animal model of 70% liver I/R injury

Male BALB/c mice (weight, 20-25 g) were used as experimental animals, maintained on a standard diet and water ad libitum, and kept in a temperature-controlled environment (20°C to 22°C) with alternating 12-hour cycles of light and dark. Six groups were studied (n = 16/group): Group I, sham group; group II, I/R group; group III, IPO+I/R group (occlusing the porta hepatis for 60 min, then treated for three cycles of 10 sec brief reperfusion consecutively, followed by a persistent reperfusion); group IV, L-NAME+sham (L-NAME, 16 mg/kg, i.v., 5 min before reperfusion); group V, L-NAME+I/R; and group VI, L-NAME+ IPO. After a midline laparatomy incision, an atraumatic vascular clip was placed on the vessels blocking the portal venous and hepatic arterial blood supply to the median and left lateral lobes of the liver, which results in approximately 70% mouse liver I/R injury. The animals were placed on a heating table to maintain core body temperature at 37°C. After 55 min ischemia, 5 min before reperfusion, L-NAME was injected through the tail vein. Sham-operated animals went through the same surgical procedure as other animals; however, hepatic vessels clip were not applied. Animals were killed at 2, 4 and 12 hours after liver I/R injury or sham surgery. Liver tissues and blood samples were taken for analysis. This study was approved by Sichuan Bioethics Committee, and all protocols were conducted under the guidelines of Animal Care and Use.

### Serum alanine aminotransferase (ALT), NO, and NOS

Blood samples were obtained at the time of sacrifice. The serum concentration of alanine aminotransferase (ALT) was measured in a clinical laboratory as markers of hepatic functional damage. The serum levels of NO and NOS were determined by using an NO and NOS Kit (Jiancheng Biotech Ltd, Nanjing, China) according to the manufacture' instructions.

### Histopathologic analysis

Tissue samples taken at the time of sacrifice after hepatic I/R injury were fixed in 10% buffered formalin solution and embedded in paraffin. Sections at 5 μm intervals were prepared and processed for H&E staining. Histological changes were scored in a blind fashion from 0 to 3 based on the degree of cytoplasmic vacuolization, sinusoidal congestion, sinusoidal derangement, and necrosis of parenchymal cells using modified Suzuki classification as described by Takeda et al [28].

### Determination of malondialdehye (MDA) level, total superoxide dismutase (SOD) activity, and nitricoxide synthase (NOS) in tissue

The involvement of ROS in I/R includes increased lipid peroxidation (LPO). LPO causes production of secondary products, among which MDA is used widely as a marker of oxidative stress. Levels of MDA in 2 hours post-ischemic livers were measured as previously described [29]. Liver samples were homogenized and trichloroacetic acid was added to the homogenate, followed by addition of TBA-water solution to the supernatant and boiling for 60 minutes. After samples were cooled down, the optical density of supernatant at 532 nm was measured. Total SOD activity was determined by monitoring the concentration of nitroblue tetrazolium, which was reduced to a water-insoluble blue formazan dye with an absorbance maximum at about 560 nm by superoxide anion generated by xanthine-xanthine oxidase as previously described [30]. Data are expressed as mean ± SD. NOS contents were assayed by using NOS assay kit (Jiancheng Biotech Ltd, Nanjing, China) according to the manufactures' instructions.

### Measurement of hepatic TNF-α and ICAM-1 mRNA levels

Total RNA was extracted from liver tissues using TRIzol reagent (Invitrogen, Carlsbad, CA). For semiquantitative PCR analysis, cDNA samples were standardized based on the content of β-actin cDNA as a housekeeping gene. RNA (1 μg) was reverse-transcribed and amplified using TaKaRa One-Step RT-PCR Kit (Takara Shuzo Co., Japan) at following RT-PCR conditions: 95°C for 2 min, 30 cycles at 95°C for 1 min, 59°C for 90 seconds, and 72°C for 2 min. Primers used in PCR reactions were as follows: TNF-α 5' primer (5'-AGCCCACGTAGCAAACCACCAA-3') and 3' primer (5'-ACACCCATTCCCTTCACAGAGCAAT-3'); ICAM-1 5' primer (5'-TGGAACTGCACGTGCTGTAT-3') and 3' primer (5'-ACCATTCTGTTCAAAAGCAG-3');

and β-actin 5' primer (5'-CTGAAGTACCCCATTGAACATGGC-3') and 3' primer (5'-CAGAGCAGTAATCTCCTTCTGCAT-3'). PCR products were stained with ethidium bromide and electrophoresed in a 1.5% agarose gel. The target bands were visualized with an ultraviolet illuminator (Gel Doc EQ) (Bio-Rad Laboratories Inc., Hercules, CA) and image analysis software (QUANTITY ONE) (Bio-Rad). The mRNA expressions of TNF-α and ICAM-1 were presented as percent of β-actin.

### Protein expression of HIF-1α, p-Akt and Akt

Proteins were extracted from hepatic tissues and quantified using the Bradford assay (Bio-Rad). Equal amounts of protein (40 μg) were separated by sodium dodecyl sulfate-polyacrylamide gel electrophoresis (SDS-PAGE). The proteins were transferred onto polyvinylidene difluoride (PVDF) membranes (Bio-Rad). After overnight blocking at 4°C, the membranes were incubated and shaken for 2 h at 37 °C with a mouse monoclonal antibody against HIF-1α (diluted 1:500, AbCam, Canbridge, UK); p-Akt (diluted 1:500, Signalway Antibody); rabbit polyclonal antibody against Akt (diluted 1:500, Signalway Antibody); followed by a secondary antibodies (diluted 1:2000, Santa Cruz, CA). The signals were detected by using an ECL kit(Millipore, Bedford, MA, USA). The membranes were re-incubated with a mouse monoclonal antibody against glyceraldehydes 3-phosphate dehydrogenase (GAPDH) (diluted 1:10,000, Santa Cruz, CA) to control for protein loading.

### Immunohistochemistry for TNF-α and ICAM-1

Tissue samples taken at the time of sacrifice after liver I/R injury were fixed in 10% buffered formalin and embedded in paraffin. Sections at 5 μm intervals were stained with primary rabbit anti-mouse mAbs against TNF-α (diluted, 1:500, Santa Cruz, CA) or ICAM-1(diluted, 1:500, Santa Cruz, CA). After incubation, the sections were incubated with a biotinylated rabbit anti-mouse IgG. Then the samples were incubated with peroxidase-labeled streptavidin. DAB solution was added to the samples, and the colorimetric reaction was allowed to proceed for 1 min. The estimates were performed by a blinded pathologist (3 to 4 sections per liver and 10 to 12 fields per section).

### Statistical analysis

All data were expressed as mean ± SD. Data were analyzed using ANOVA for multiple comparisons. Analysis between two groups was performed using unpaired Student's t test (two-tailed) where ANOVA indicated significance for the multiple comparison. P values of less than 0.05 were considered as significant differences.

## Results

### Physiological function of IPO in hepatic I/R injury

**Figure 1:**
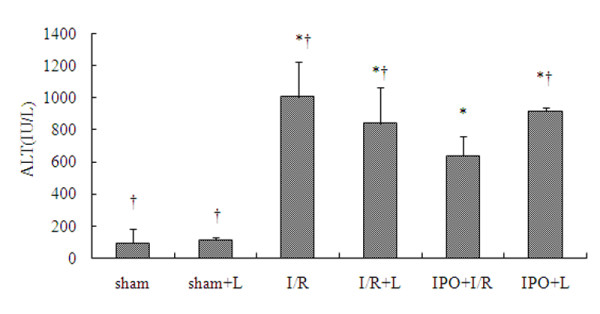
**ALT levels after reperfusion**. After 60 minutes of ischemia and 2 hours of reperfusion, serum levels of ALT were determined. Compared with sham-operated control mice, I/R mice showed significant increases in ALT. The post-treatment of IPO significantly reduced all serum levels of ALT compared to I/R group. "+L" means "+L-NAME". For all groups, n = 8. * p < 0.05 compared to sham group. † p < 0.05 compared to IPO+I/R group.

To determine if IPO was able to attenuate I/R injury, 3 cycles of 10s of reperfusion followed by 10s ischemia immediately after 60 min ischemia of the medium and left liver lobes were applied to the IPO+I/R group. Serum levels of ALT were measured after 2 h of reperfusion following 60 min of ischemia and were significantly different among the groups. Compared with sham-operated control mice, I/R mice showed significant increases in ALT. IPO treatment significantly reduced all serum levels of ALT compared to I/R group (Figure 1). Subsequent determination of transaminases levels at 4, 12 h of reperfusion showed maintained low values in mice post-treated with IPO but high levels in I/R group (data not shown).

### Protective effect of IPO on the liver tissue from I/R injury

**Figure 2:**
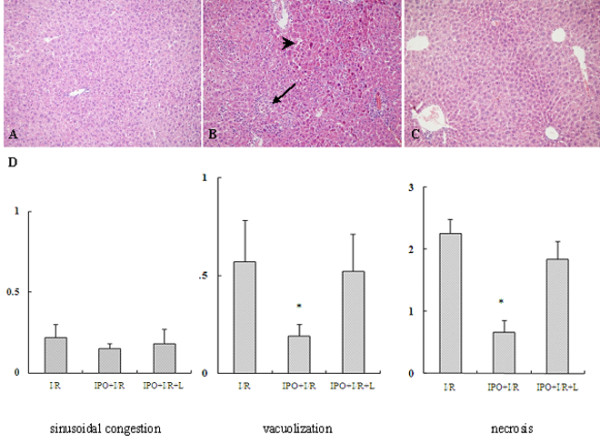
**Hepatic histological changes in mice subjected to I/R**. (A): sham, (B): I/R, (C): IPO+I/R. Hematoxylin-eosin-stained liver sections from animals undergoing 60 minutes ischemia and 2 hours following reperfusion (Original magnification: ×200). Decreased hepatic necrosis is seen in the IPO+I/R group compared to the nontreated I/R group. Images are representative liver sections from eight mice per group. Black arrow shows the infiltrated neutrophils and black arrow head shows hepatic cellular necrosis in Figure 2B. (D): Histological scores for sinusoidal congestion, cytoplasmic vacuolization, and hepatocyte necrosis were obtained via analysis of hematoxylin-eosin staining. Data are expressed as the mean ± SD of 8 animals per group. * p < 0.05 compared with I/R group.

To further confirm the protective effect of IPO on hepatic I/R injury, sections of the liver obtained from the ischemic lobe at 2 h after reperfusion were evaluated for histopathological analysis. Compared with sham-operated control group(Figure 2A), I/R mice liver tissue showed significant cytoplasmic vacuolization, sinusoidal congestion, extensive hepatic cellular necrosis and massive cellular infiltration (Figure 2B). However, the parenchymal appearance was near normal in IPO+I/R group. Mild cellular infiltration, few necrosis as well as comparatively preserved lobular architecture were seen in the liver treated with IPO (Figure 2C). In the evaluation of the histological features of I/R injury, the IPO+I/R group had significantly lower scores of cytoplasmic vacuolization and massive necrosis compared with the I/R group (Figure 2D). L-NAME abolished the protective effect of IPO post-treatment with increased cytoplasmic vacuolization and hepatocellular necrosis (Figure 2D).

### IPO reduces oxidative stress in liver tissues

**Figure 3:**
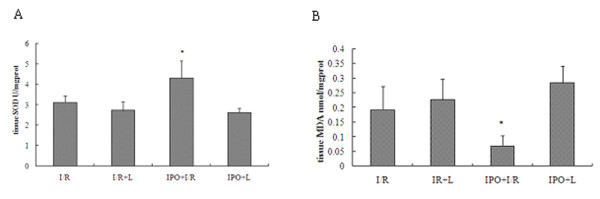
**Effects of IPO on SOD (A) and MDA (B) levels in liver tissues**. To assess the effect of IPO on oxidative stress after liver I/R, MDA and activity of SOD were measured. Hepatic 60 minutes ischemia and 2 hours of reperfusion caused substantial increase in liver MDA levels and marked decrease in liver SOD activity compared with IPO+I/R group. For all groups, n = 8. *Significant at p < 0.05 when compared with I/R group.

To assess the effect of IPO on oxidative stress after liver I/R, MDA and activity of superoxide dismutase (SOD) were measured. Hepatic 60 min ischemia and 2 hours of reperfusion caused substantial increase in liver MDA levels and marked decrease in liver SOD activity compared with IPO+I/R group (Figure 3). In the post-treatment of IPO, the liver MDA content reduced 64.11% and liver SOD activity was elevated by 27.68%.

### IPO increases NO, NOS in serum and in liver tissues

**Figure 4:**
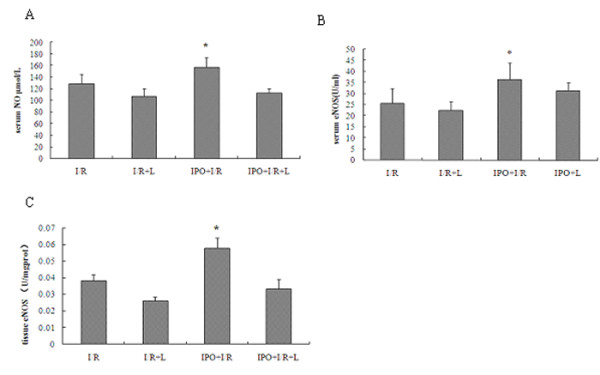
**Effects of IPO on NO in serum (A), eNOS in serum (B) and in liver tissues (C)**. To determine whether IPO have protective role through NO-mediated production, the contents of NO and NOS were detected. Compared to I/R group, IPO post-treatment markedly induced NO and eNOS production in serum and in liver tissues. For all groups, n = 8. * p < 0.05 compared with I/R group.

To determine whether IPO have protective role through NO-mediated production, we detected the contents of nitric oxide (NO) and nitric oxide synthase (NOS). Hepatic 60 min ischemia and 2 hours of reperfusion markedly reduced both the serum levels of NO, total NOS (TNOS), endothelial NOS (eNOS), iNOS, and production of TNOS, eNOS, iNOS in liver tissues. Compared to I/R group, IPO post-treatment markedly induced NO, eNOS in serum (U/ml: 22.21 ± 1.13 vs. 36.33 ± 7.57), and eNOS in tissue (U/mgprot: 0.038 ± 0.004 vs. 0.058 ± 0.006; I/R vs. IPO) (Figure 4), while no significant difference was found in TNOS [serum(U/ml): 38.514 ± 4.074 vs. 46.147 ± 7.045, tissue(U/mgprot): 0.107 ± 0.045 vs. 0.131 ± 0.038; I/R vs. IPO] both in serum and tissues, and iNOS [serum(U/ml): 12.971 ± 3.055 vs. 10.817 ± 2.116, tissue(U/mgprot): 0.069 ± 0.018 vs. 0.073 ± 0.014; I/R vs. IPO] both in serum and tissues between I/R and IPO+I/R group. Although no significant difference was found in TNOS between I/R and IPO+I/R group, some trends of higher TNOS levels could be seen in the IPO+I/R group. In L-NAME+ IPO and L-NAME+ I/R groups, the serum levels of NO, TNOS, eNOS and iNOS, production of TNOS, eNOS, iNOS in liver tissues were all decreased. These findings suggest that IPO have protective role partially through up-regulating NO and iNOS.

### IPO induce HIF-1α and p-Akt expression in liver tissues and modulates I/R-induced inflammatory signaling cascades

**Figure 5:**
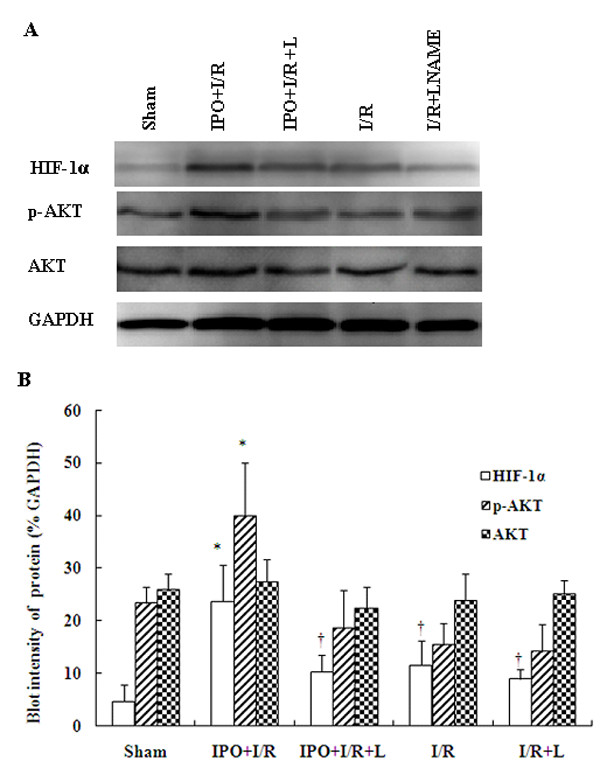
**Expression of HIF-1alpha, p-Akt and Akt by Western blot**. The expression of HIF-1alpha, p-Akt and Akt were detected in liver tissues by western blot analysis. The blot shown is representative of three different experiments with similar results (A). Lain 1-5: sham; IPO+I/R; IPO+I/R+L-NAME; I/R; I/R+L-NAME. The expression of the housekeeping gene, glyceraldehydes 3-phosphate dehydrogenase (GAPDH), served as a control. The expression of HIF-1alpha, and p-Akt were significantly higher in the liver tissues with IPO+I/R group than I/R group, and the signals were decreased in liver tissues with L-NAME (16 mg/kg) pre-treatment. HIF-1alpha, p-Akt and Akt proteins were calculated by densitometry relative to GAPDH, and the results were expressed as ratios after normalization at 100% of the control (B). Data are mean ± SD from three separate experiments. * p < 0.05 compared with other groups. † p < 0.05 compared to sham group.

To further assess whether the NO-mediated production is associated with HIF-1alpha, we measured the protein expressions of HIF-1alpha and p-Akt by western blot analysis. Western blot analysis results showed that the contents of HIF-1α in liver tissues with IPO post-treated mice were significantly higher than those in the I/R group (Figure 5). Reports have shown that PI3K signaling pathway is involved in HIF-1α up-regulation in the relevant experiments[19,31]. So we also determined whether IPO altered liver I/R-induced PI3K signaling pathway activation. And Figure 5 shows changes in phosphorylation of Akt upon reperfusion. The ratios of p-Akt and Akt in sham, IPO+I/R, IPO+I/R+L, I/R, I/R+L groups were as follows: 0.91 ± 0.31, 14.53 ± 2.88, 0.84 ± 0.15, 0.64 ± 0.15, 0.57 ± 0.12. So IPO post-treatment markedly enhanced Akt phosphorylation at reperfusion compared to other group (Figure 5), corroborating the role of the PI3K/Akt pathway in the action of IPO.

### IPO reduces TNF-α and ICAM-1 mRNA in liver tissues

**Figure 6:**
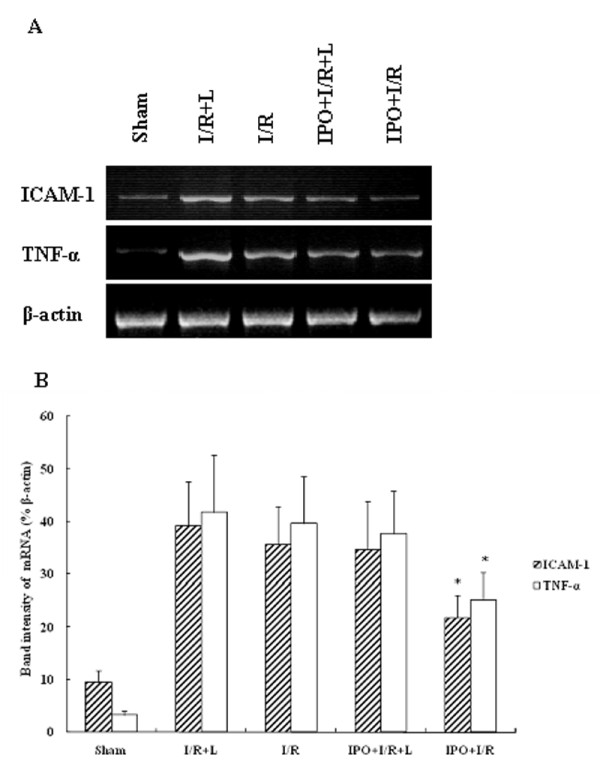
**RT-PCR product of TNF-α and ICAM-1 using template RNA extracted from 4 h post-ischemic liver tissues (A)**. IPO significantly abrogated liver warm I/R-induced increases in TNF-α and ICAM-1 mRNA expression. Lain 1-5: sham; I/R+L-NAME; I/R; IPO+I/R+L-NAME; IPO+I/R. Representative experiments of three are shown in each case. The mRNA band intensities of TNF-α and ICAM-1 in sham, I/R+L-NAME, I/R, IPO+I/R+L-NAME, IPO+I/R groups were compared as indicated (B). (n = 8). Data are mean ± SD from three separate experiments. * p < 0.05 compared with other groups.

To determine the expressions of proinflammatory mediators and adhesion molecules, mRNA transcripts for TNF-α and ICAM-1 were assessed. Liver I/R remarkably increased mRNA expression of TNF-α and ICAM-1. IPO significantly abrogated liver warm I/R-induced increases in TNF-α and ICAM-1 mRNA expression (Figure 6A). L-NAME treatment did not decrease the up-regulation of TNF-α and ICAM-1 mRNA expression. The comparison of band intensity ratios of ICAM-1 to β-actin demonstrated that IPO treatment effectively suppressed the TNF-α and ICAM-1 mRNA expression induced by I/R injury (Figure 6B).

### IPO reduces TNF-α and ICAM-1 protein in liver tissues

**Figure 7:**
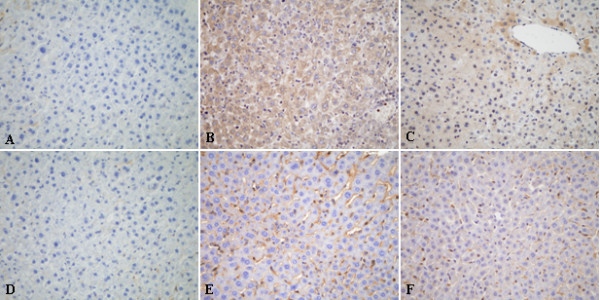
**Immunohistochemical assay of TNF-α (A, B, C) and ICAM-1(D, E, F) on 4 h post-ischemic liver tissue**. In the IPO+I/R group, hepatic I/R-induced increases in TNF-α and ICAM-1 expression were dramatically suppressed. (A, D): sham group, (B, E): I/R group, (C, F): IPO+I/R group. Original magnification: ×400.

To further determine the protein expression changes of TNF-α and ICAM-1 induced by IPO, we detected these protein expressions by immunohistochemical assay. The over-expressions of TNF-α and ICAM-1 on liver tissues after 4 h of reperfusion were detected (Figure 7B, E). In IPO+I/R group, hepatic I/R-induced increases in TNF-α and ICAM-1 expression were dramatically suppressed (Figure 7C, F). While the up-regulation of TNF-α and ICAM-1 protein expressions were not decreased in the L-NAME+ IPO group. These findings suggest that IPO have a role in modulating the inflammatory process.

### L-NAME abolishes the hepatic protection by IPO

To further confirm the NO protection against I/R injury, we also applied a non-selective NOS inhibitor, L-NAME, in the experimental groups. And we found the treatment with L-NAME almost completely abolished the liver protective effect of IPO against I/R-induced hepatic dysfunction (Figures 1, 2, 3, 4, 5, 6).

## Discussions

We investigated the potential protective mechanism of IPO on hepatic warm I/R injury. It was observed that IPO post-treatment could effectively attenuate liver injury in a model of mice hepatic warm I/R. The protective effect of IPO was associated with an enhanced, sustained NO generation at reperfusion that was abrogated by NOS inhibition. IPO also increased expression of HIF-1α and phosphorylation of the survival kinase Akt following I/R while inhibiting ROS production, suppressing the over-expression of proinflammatory mediators and adhesion molecules. These results suggest that IPO protects liver from I/R injury, at least in part, by increasing HIF-1α and p-Akt, and suppressing ROS production, which lead to the maintenance of an elevated level of NO.

A series of studies have demonstrated that IPO effectively protects against I/R injuries through NO-mediated production [17,18]. Unfortunately, little is known about the more detailed protective mechanism of IPO on liver I/R injury. So we demonstrated that IPO, 3 cycles of 10 s of reperfusion followed by 10 s ischemia immediately after 60 min ischemia, exhibited significant protection to the mice liver from I/R injury, as assessed by liver function tests and histology. IPO post-treatment significantly reduced serum levels of ALT, and contributed to significantly lower scores of cytoplasmic vacuolization and massive necrosis compared with the I/R group. L-NAME treatment almost completely abolished the liver protective effect of IPO against I/R injury morphologically and functionally.

It has been demonstrated by several studies that NO could attenuate I/R injury of different organs [15,16]. Nitric oxide can also cross biological membranes and travel significant distances in cells and tissues [32]. Lang et al recently reported that inhaled NO accelerates restoration of liver function in adults following liver transplantation [33]. It has also been reported that IPO could stimulate production of NO [17,18], so we determined if IPO would protect liver against liver I/R injury through NO-mediated production. We observed the changes of NO levels in serum and tissues, as well as NOS. Until now, three different kinds of NOS have been identified. Previous study has demonstrated that nNOS were expressed in liver tissue of mouse, but it has been reported that nNOS is mainly involved in neuronal signaling and it does not participate in the events involved during I/R [34]. So we detected the serum levels of NO, TNOS, eNOS, iNOS and production of TNOS, eNOS, iNOS in liver tissues.

Hepatic I/R significantly reduced both the serum levels of NO, TNOS, eNOS, iNOS and production of TNOS, eNOS, iNOS in liver tissues. Increased NO, eNOS in serum and eNOS in tissue (Figure 4) were found in IPO+I/R group, while no significant differences were found in TNOS and iNOS in serum and tissues between I/R and IPO+I/R group. L-NAME decreased the serum levels of NO, TNOS, eNOS and iNOS, production of TNOS, eNOS, iNOS in liver tissues. eNOS was reported to play a beneficial role against I/R injury. It was found that eNOS could lead to amelioration of I/R-induced liver injury [35,36] and protect against renal I/R injury [37]. eNOS over-expression also could lead to reduced infarct sizes after cardiac I/R injury [36,37]. NO production by eNOS seems to be of central importance in ischemic injury [38,39]. It has been reported that eNOS-derived NO production constitutes a promising therapeutic approach to prevent myocardial I/R injury [40]. Our results showed that increased eNOS levels both in serum and tissue using assay kits. So it increased both locally and systemically, and that might contribute to NO production and liver protection. These findings support our hypothesis that the IPO elevates NO and eNOS levels, which in turn reduces or compensates the I/R-induced hepatic injury.

ROS play a critical role in the I/R injury. After warm ischemia, ROS were produced at the moment of reperfusion and promoted the adhesion of leukocytes to microvascular endothelium [41]. Our study showed that IPO post-treatment reversed the increase of MDA levels to a considerable extent, thereby confirming its antioxidant role in I/R. Furthermore, we showed that SOD activity significantly increased in IPO+I/R group. Total SOD activity is decreased following I/R injury [42], and the decrease would render the tissue susceptible to oxidant injury. Therefore, the elevated SOD activity induced by IPO post-treatment may contribute to reduce superoxide radicals following liver I/R. Our results indicated IPO may reduce the oxidative stress caused by hepatic warm I/R injury and attenuate subsequent organ damages (Figure 3). It has been shown that NO may augment antioxidant protection by forming intracellular antioxidants (nitrosothiols and glutathione) [43] and by decreasing ROS release through inhibition of NADPH oxidase activity [44]. ROS was also significantly reduced in mice treated with the eNOS enhancer [40]. In turn, bioavailability of NO can be reduced by oxidative inactivation by excessive production of the superoxide anion. Increased generation of superoxide may inhibit the physiological functions of NO [45]. In contrast, SOD can also rapidly scavenge superoxide (O2-) and prolong the vasorelaxant effects of NO [46]. NO responses can be restored by the addition of superoxide dismutase (SOD). So in our study, elevated NO induced by IPO might contribute to reducing ROS release, and also decreased MDA and increased SOD by IPO could contribute to the beneficial effect of NO.

Several reports have shown that HIF-1 activation might attenuate I/R injury [47-49]. Since HIF-1 can upregulate genes intimately involved in ischemic preconditioning (e.g., VEGF [50], and HO-1[51], it becomes an attractive molecular target to limit ischemic or postischemic tissue injury. In our study, we found that IPO post-treatment could up-regulate the expression of HIF-1α. The contents of HIF-1α in liver tissues in IPO+I/R group were significantly higher than those in I/R group. It has been shown that NO could influence the levels of HIF-1α in complex ways. NO concentration has a strong influence on whether HIF-1α is stabilized under aerobic conditions [52]. PHD is active under normal oxygen supply and can hydroxylate HIF-1α [53]. Under normoxia, NO can block PHD activity by interacting with enzyme bound Fe^2+^, directly attenuate hydroxylation of HIF-1α [54] and accumulate HIF-1α. Exposure to NO has been shown to nitrosylate thiols in the HIF-1α protein leading to HIF-1α stabilization [55,56]. NO can also promote binding of HIF-1α to hypoxia response elements (HREs) in HIF-1α target genes and act as a transcriptional co-activator [57]. NO can act as a diffusible, paracrine messenger to elicit a functional HIF-1 response [58,59]. In turn, unregulated VEGF induced by HIF-1 can activate eNOS in vascular endothelial cells through adenylate cyclase (AC)-protein kinase A (PKA), phosphoinositide 3-kinase (PI3K)-Akt pathways [60], and HIF-1 has been reported to be able to improve the actions of NO [61]. So in our study, elevated NO levels by IPO post-treatment at 2 h after reperfusion contributed to increasing HIF-1α stability, and in turn, up-regulated expression of HIF-1α induced by IPO might also increase the levels of eNOS and NO.

PI3K and its downstream regulated protein Akt as well as eNOS are known to play important roles in survival against ischemia/reperfusion injury. Studies have shown that NO can upregulate the rate of HIF-1α synthesis by activating the PI3K-MAPK (mitogen-activated protein kinase) pathway [19,20]. It was found that the NO donor NOC18 treatment could induce phosphorylation of Akt, HIF-1α protein expression and HIF-1 transcriptional activation [20]. In our study, western blot analysis results showed that IPO post-treatment could markedly enhance Akt phosphorylation at 2 h after reperfusion compared to control group, and p-Akt was markedly decreased after using L-NAME. So increased NO levels induced by IPO might help in increasing the expression of p-Akt, and then upregulating the rate of HIF-1α synthesis. In turn, Akt has been shown to increase the formation of NO, specifically via the activation of eNOS [62]. Unregulated VEGF induced by HIF-1 can activate eNOS also through PI3K/Akt pathway, and increased the NO production. PI3K is a redox-sensitive kinase; thus, it may be activated through changes in intracellular ROS levels, leading to eNOS activation and increased NO release [63]. It was reported that ischemic postconditioning's protection involves adenosine receptors and requires PI3-kinase activation [24]. It has been shown that inhibiting PI3K using LY294002 or Wortmannin (Wort) completely abolished IPO-induced protection, so IPO could protect the myocardium by activating the PI3K/Akt pathway [23]. And it was also reported that the PI3K inhibitor LY294002 enlarged infarct in ischemic postconditioned rats, and LY294002 could also abolish the protective effects of IPO on both disease models and healthy hearts, so PI3K/Akt pathway contributes to postconditioning's protection [64,65]. These results also suggested that the PI3K/Akt pathway could play a role in the protective action of liver IPO.

Studies have shown TNF-α could activate neutrophils to release inflammatory mediators and play an important role in I/R injury. TNF-α also caused overexpression of adhesion molecules on both endothelial cells and leukocytes [66], and increased neutrophils aggregation and adhesion to endothelial cells. In this study, the I/R-induced increases in hepatic levels of TNF-α was inhibited in IPO+I/R group (Figure 7) and this effect was confirmed by RT-PCR analysis on TNF-α mRNA in liver tissues (Figure 6). The I/R-induced hepatic accumulation of neutrophils was also decreased following IPO treatment (Figure 2). Thus, inhibition of TNF-α production may prevent the subsequent neutrophils activation. Accumulating evidence indicates that ischemia alone may induce TNF-α mRNA and protein via the generation of ROS [67]. Activation of oxidant-sensitive enzymes involved in TNF-α production represents an additional mechanism by which oxidant stress induces cellular damage. ICAM-1 is also important in the pathogenesis of I/R injury. Hydrogen peroxide can also induce endothelial ICAM-1 through activation of transcriptional factors, such as nuclear transcription factor κB (NF-κB) [68]. Our results showed that increased expression of ICAM-1 was observed 4 h post-reperfusion in untreated mice and IPO effectively suppressed the overexpression of ICAM-1 on liver tissue and abrogated hepatic I/R-induced increase in ICAM-1 mRNA expression (Figure 6). Therefore, the inhibition of I/R-induced increases of ROS following IPO treatment may help in reducing the overexpression of TNF-α and ICAM-1.

Nitric oxide (NO) has been reported to decrease endothelial ICAM-1 mRNA and surface expression, which results in reduction in PMNs adhesion to endothelium stimulated by TNF-α [69]. One mechanism by which NO may modulate the inflammatory process is via its interaction with the Rel/NF-κB family of transcription factors. In the current study, we found that IPO posttreatment significantly reduced hepatic ICAM-1 mRNA levels during early reperfusion periods, and suppressed neutrophil accumulation in liver. These findings are consistent with previous reports that inhibition of NO synthesis increased ICAM-1 expression and enhanced neutrophil-dependent reperfusion injury in hepatic warm I/R injury [70] and that NO enhancement attenuated neutrophil infiltration and hepatic warm I/R injury [71]. Therefore, up-regulated NO by IPO post-treatment might also have a role in modulate the infammatory process by decreasing the expression of TNF-α and ICAM-1.

## Conclusions

In conclusion, our investigations demonstrated that IPO, 3 cycles of 10 s of reperfusion followed by 10 s ischemia, resulted in protection in liver warm I/R injury which was associated with increases in NO, eNOS, SOD, p-Akt and HIF-1α, and decrease in ROS, TNF-α and ICAM-1. IPO induced protection was abrogated in the presence of the NO inhibitor L-NAME. The increased NO concentration produced a cytoprotective environment, resulting in reduced cell death and restoration of hepatic function following reperfusion. Thus, the protection conferred by IPO appears to be mediated by increased NO and HIF-1α productions during reperfusion via the activation of Akt and inhibition of ROS. These findings suggested IPO might have the therapeutic potential through Akt-eNOS-NO-HIF pathway for the better management of liver warm I/R injury.

## Competing interests

The authors declare that they have no competing interests.

## Authors' contributions

YG, TY and YZ conceived the study, established the design and carried out the experimental work. FL, DL and QL performed the animal model and relevant experiments. YL participated in the data analysis and provided critical comments on the study design and manuscript. LF contributed to the design and coordination of the study, and helped to draft the final version of this manuscript. All authors read and approved the final manuscript.

## References

[B1] Carini R Albano E Recent insights on the mechanisms of liver preconditioning Gastroenterology 2003 125 1480 1491 10.1016/j.gastro.2003.05.005 14598265

[B2] Oshima Y Fujio Y Nakanishi T Itoh N Yamamoto Y Negoro S Tanaka K Kishimoto T Kawase I Azuma J STAT3 mediates cardioprotection against ischemia/reperfusion injury through metallothionein induction in the heart Cardiovasc Res 2005 65 428 435 10.1016/j.cardiores.2004.10.021 15639482

[B3] Zhao ZQ Corvera JS Halkos ME Kerendi F Wang NP Guyton RA Vinten-Johansen J Inhibition of myocardial injury by ischemic postconditioning during reperfusion: comparison with ischemic preconditioning Am J Physiol Heart Circ Physiol 2003 285 579 588 10.1152/ajpheart.01064.2002 12860564

[B4] Cai M Li Y Xu Y Swartz HM Chen CL Chen YR He G Endothelial NOS activity and myocardial oxygen metabolism define the salvageable ischemic time window for ischemic postconditioning Am J Physiol Heart Circ Physio 2011 300 H1069 1077 10.1152/ajpheart.00694.2010 PMC3064316 21217066

[B5] Lønborg J Kelbaek H Vejlstrup N Jørgensen E Helqvist S Saunamäki K Clemmensen P Holmvang L Treiman M Jensen JS Engstrøm T Cardioprotective effects of ischemic postconditioning in patients treated with primary percutaneous coronary intervention, evaluated by magnetic resonance Circ Cardiovasc Interv 2010 3 34 41 10.1161/CIRCINTERVENTIONS.109.905521 20118154

[B6] Penna C Tullio F Moro F Folino A Merlino A Pagliaro P Effects of a protocol of ischemic postconditioning and/or captopril in hearts of normotensive and hypertensive rats Basic Res Cardiol 2010 105 181 192 10.1007/s00395-009-0075-6 20012872

[B7] Rehni AK Singh N Role of phosphoinositide 3-kinase in ischemic postconditioning-induced attenuation of cerebral ischemia-evoked behavioral deficits in mice Pharmacol Rep 2007 59 192 198 17556797

[B8] Liu X Chen H Zhan B Xing B Zhou J Zhu H Chen Z Attenuation of reperfusion injury by renal ischemic postconditioning: the role of NO Biochem Biophys Res Commun 2007 359 628 634 10.1016/j.bbrc.2007.05.129 17548062

[B9] Chen H Xing B Liu X Zhan B Zhou J Zhu H Chen Z Ischemic postconditioning inhibits apoptosis after renal ischemia/reperfusion injury in rat Transpl Int 2008 21 364 371 10.1111/j.1432-2277.2007.00606.x 18069925

[B10] Huang H Zhang L Wang Y Yao J Weng H Wu H Chen Z Liu J Effect of ischemic post-conditioning on spinal cord ischemic-reperfusion injury in rabbits Can J Anaesth 2007 54 42 48 10.1007/BF03021898 17197467

[B11] Santos CHM Gomes OM Pontes JCDV Miiji LNO Bispo MA The ischemic preconditioning and postconditioning effect on the intestinal mucosa of rats undergoing mesenteric ischemia/reperfusion process Acta Cir Bras 2008 23 22 28 18278389 10.1590/s0102-86502008000100005

[B12] Santos CH Pontes JC Miiji LN Nakamura DI Galhardo CA Aguena SM Postconditioning effect in the hepatic ischemia and reperfusion in rats Acta Cir Bra 2010 25 163 168 10.1590/s0102-86502010000200008 20305883

[B13] Zhang WX Yin W Zhang L Wang LH Bao L Tuo HF Zhou LF Wang CC Preconditioning and postconditioning reduce hepatic ischemia-reperfusion injury in rats Hepatobiliary Pancreat Dis Int 2009 8 586 590 20007074

[B14] Teixeira AR Molan NT Kubrusly MS Bellodi-Privato M Coelho AM Leite KR Machado MA Bacchella T Machado MC Postconditioning ameliorates lipid peroxidation in liver ischemia-reperfusion injury in rats Acta Cir Bras 2009 24 52 56 19169543 10.1590/s0102-86502009000100011

[B15] Jalowy A Schulz R Heusch G AT1 receptor blockade in experimental myocardial ischemia/reperfusion J Am Soc Nephrol 1999 10 S129 136 9892153

[B16] Kubota I Han X Opel DJ Zhao YY Baliga R Huang P Fishman MC Shannon RP Michel T Kelly RA Increased susceptibility to development of triggered activity in myocytes from mice with targeted disruption of endothelial nitric oxide synthase J Mol Cell Cardiol 2000 32 1239 1248 10.1006/jmcc.2000.1158 10860766

[B17] Yang XM Proctor JB Cui L Multiple, brief coronary occlusions during early reperfusion protect rabbit hearts by targeting cell signaling pathways J Am Coll Cardiol 2004 44 1103 1110 10.1016/j.jacc.2004.05.060 15337225

[B18] Liu X Chen H Zhan B Xing B Zhou J Zhu H Chen Z Attenuation of reperfusion injury by renal ischemic postconditioning: the role of NO Biochem Biophys Res Commun 2007 359 628 634 10.1016/j.bbrc.2007.05.129 17548062

[B19] Kasuno K Takabuchi S Fukuda K Kizaka-Kondoh S Yodoi J Adachi T Semenza GL Hirota K Nitric oxide induces hypoxia-inducible factor 1 activation that is dependent on MAPK and phosphatidylinositol 3-kinase signaling J Biol Chem 2004 279 2550 2558 14600153 10.1074/jbc.M308197200

[B20] Sandau KB Faus HG Brune B Induction of hypoxia-inducible-factor 1 by nitric oxide is mediated via the PI 3K pathway Biochem Biophys Res Commun 2000 278 263 267 10.1006/bbrc.2000.3789 11071882

[B21] Zhong Z Ramshesh VK Rehman H Currin RT Sridharan V Theruvath TP Kim I Wright GL Lemasters JJ Activation of the oxygen-sensing signal cascade prevents mitochondrial injury after mouse liver ischemia-reperfusion Am J Physiol Gastrointest Liver Physiol 2008 295 G823 832 10.1152/ajpgi.90287.2008 18772364 PMC2575910

[B22] Alchera E Tacchini L Imarisio C Dal Ponte C De Ponti C Gammella E Cairo G Albano E Carini R Adenosine-dependent activation of hypoxia-inducible factor-1 induces late preconditioning in liver cells Hepatology 2008 48 230 239 10.1002/hep.22249 18506850

[B23] Tsang A Hausenloy DJ Mocanu MM Postconditioning: a form of "modified reperfusion" protects the myocardium by activating the phosphatidylinositol 3-kinase-akt pathway Circ Res 2004 95 230 232 10.1161/01.RES.0000138303.76488.fe 15242972

[B24] Yang XM Philipp S Downey JM Postconditioning's protection is not dependent on circulating blood factors or cells but involves adenosine receptors and requires PI3-kinase and guanylyl cyclase activation Bas Res Cardiol 2005 100 57 63 10.1007/s00395-004-0498-4 15614590

[B25] Shao ZH Wojcik KR Dossumbekova A Hsu C Mehendale SR Li CQ Qin Y Sharp WW Chang WT Hamann KJ Yuan CS Hoek TL Grape seed proanthocyanidins protect cardiomyocytes from ischemia and reperfusion injury via Akt-NOS signaling J Cell Biochem 2009 107 697 705 10.1002/jcb.22170 19388003

[B26] Gao F Gao E Yue TL Ohlstein EH Lopez BL Christopher TA Ma XL Nitric oxide mediates the antiapoptotic effect of insulin in myocardial ischemia-reperfusion: The roles of PI3-kinase, Akt, and endothelial nitric oxide synthase phosphorylation Circulation 2002 105 1497 1502 10.1161/01.CIR.0000012529.00367.0F 11914261

[B27] Li J Zhang H Wu F Nan Y Ma H Guo W Wang H Ren J Das UN Gao F Insulin inhibits tumor necrosis factor-alpha induction in myocardial ischemia/reperfusion: Role of Akt and endothelial nitric oxide synthase phosphorylation Crit Care Med 2008 36 1551 1558 10.1097/CCM.0b013e3181782335 18434880

[B28] Takeda K Jin MB Fujita M Fukai M Sakurai T Nakayama M Taniguchi M Suzuki T Shimamura T Furukawa H Todo S A novel inhibitor of Rho-associated protein kinase, Y-27632, ameliorates hepatic ischemia and reperfusion injury in rats Surgery 2003 133 197 206 10.1067/msy.2003.59 12605181

[B29] Peralta C Perales JC Bartrons R Mitchell C Gilgenkrantz H Xaus C The combination of ischemic preconditioning and liver Bcl-2 overexpression is a suitable strategy to prevent liver and lung damage after hepatic ischemia-reperfusion Am J Pathol 2002 160 2111 2122 10.1016/S0002-9440(10)61160-1 12057915 PMC1850813

[B30] Sun Y Oberley LW Li Y A simple method for clinical assay of superoxide dismutase Clin Chem 1988 34 497 500 3349599

[B31] Lu DY Liou HC Tang CH Fu WM Hypoxia-induced iNOS expression in microglia is regulated by the PI3-kinase/Akt/mTOR signaling pathway and activation of hypoxia inducible factor-1alpha Biochem Pharmacol 2006 72 992 1000 10.1016/j.bcp.2006.06.038 16919605

[B32] Wood J Garthwaite J Models of the diffusional spread of nitric oxide: implications for neural nitric oxide signalling and its pharmacological properties Neuropharmacology 1994 33 1235 1244 10.1016/0028-3908(94)90022-1 7870284

[B33] Lang JD Jr Teng X Chumley P Crawford JH Isbell TS Chacko BK Liu Y Jhala N Crowe DR Smith AB Cross RC Frenette L Kelley EE Wilhite DW Hall CR Page GP Fallon MB Bynon JS Eckhoff DE Patel RP Inhaled NO accelerates restoration of liver function in adults following orthotopic liver transplantation J Clin Invest 2007 117 2583 2591 10.1172/JCI31892 17717604 PMC1950460

[B34] Mathie RT Mathie RT, Griffith TM The hepatic haemodynamic effects of nitric oxide In the haemodynamic effects of nitric oxide 1999 1 London: Imperial College Press 22 51

[B35] Harada H Pavlick KP Hines IN Lefer DJ Hoffman JM Bharwani S Wolf RE Grisham MB Sexual dimorphism in reduced-size liver ischemia and reperfusion injury in mice: Role of endothelial cell nitric oxide synthase Proc Natl Acad Sci USA 2003 100 739 744 10.1073/pnas.0235680100 12522262 PMC141066

[B36] Hines IN Harada H Bharwani S Pavlick KP Hoffman JM Grisham MB Enhanced post-ischemic liver injury in iNOS-deficient mice: a cautionary note Biochem Biophys Res Commun 2001 284 972 976 10.1006/bbrc.2001.5069 11409889

[B37] Milsom AB Patel NS Mazzon E Tripatara P Storey A Mota-Filipe H Sepodes B Webb AJ Cuzzocrea S Hobbs AJ Thiemermann C Ahluwalia A Role for endothelial nitric oxide synthase in nitrite-induced protection against renal ischemia-reperfusion injury in mice Nitric Oxide 2010 22 141 148 10.1016/j.niox.2009.10.010 19892029

[B38] Jones SP Greer JJ Kakkar AK Ware PD Turnage RH Hicks M van Haperen R de Crom R Kawashima S Yokoyama M Lefer DJ Endothelial nitric oxide synthase overexpression attenuates myocardial reperfusion injury Am J Physiol Heart Circ Physiol 2004 286 H276 H282 12969888 10.1152/ajpheart.00129.2003

[B39] Sharp BR Jones SP Rimmer DM Lefer DJ Differential response to myocardial reperfusion injury in eNOS-deficient mice Am J Physiol Heart Circ Physiol 2002 282 H2422 H2426 12003854 10.1152/ajpheart.00855.2001

[B40] Frantz S Adamek A Fraccarollo D Tillmanns J Widder JD Dienesch C Schäfer A Podolskaya A Held M Ruetten H Ertl G Bauersachs J The eNOS enhancer AVE 9488: a novel cardioprotectant against ischemia reperfusion injury Basic Res Cardiol 2009 104 773 779 10.1007/s00395-009-0041-3 19548059

[B41] Granger DN Benoit JN Suzuki M Grisham MB Leucocyte adherence to venular endothelium during ischemia-reperfusion Am J Physiol 1989 257 G683 688 2596604 10.1152/ajpgi.1989.257.5.G683

[B42] Singh I Gulati S Orak JK Singh AK Expression of antioxidant enzymes in rat kidney during ischemia-reperfusion injury Mol Cell Biochem 1993 125 97 104 10.1007/BF00936438 8283974

[B43] Ronson RS Nakamura M Vinten-Johansen J The cardiovascular effects and implications of peroxynitrite Cardiovasc Res 1999 44 47 59 10.1016/S0008-6363(99)00184-4 10615389

[B44] Fujii H Ichimori K Hoshiai K Nakazawa H Nitric oxide inactivates NADPH oxidase in pig neutrophils by inhibiting its assembling process J Biol Chem 1997 272 32773 32778 10.1074/jbc.272.52.32773 9407051

[B45] Kojda G Harrison D Interactions between NO and reactive oxygen species: pathophysiological importance in atherosclerosis, hypertension, diabetes and heart failure Cardiovascular Research 1999 43 562 571 10.1016/S0008-6363(99)00169-8 10690328

[B46] Murphy ME Sies H Reversible conversion of nitroxyl anion to nitric oxide by superoxide dismutase Proc Natl Acad Sci USA 1991 88 10860 10864 10.1073/pnas.88.23.10860 1961756 PMC53031

[B47] Ockaili R Natarajan R Salloum F Fisher BJ Jones D Fowler AA Kukreja RC HIF-1 activation attenuates post-ischemic myocardial injury: a role for heme oxygenase-1 in modulating microvascular chemokine generation Am J Physiol Heart Circ Physiol 2005 289 H542 548 10.1152/ajpheart.00089.2005 15805230

[B48] Xi L Taher M Yin C Salloum F Kukreja RC Cobalt chloride induces delayed cardiac preconditioning in mice through selective activation of HIF-1alpha and AP-1 and iNOS signaling Am J Physiol Heart Circ Physiol 2004 287 H2369 2375 10.1152/ajpheart.00422.2004 15284066

[B49] Cai Z Manalo DJ Wei G Rodriguez ER Fox-Talbot K Lu H Zweier JL Semenza GL Hearts from rodents exposed to intermittent hypoxia or erythropoietin are protected against ischemia-reperfusion injury Circulation 2003 108 79 85 10.1161/01.CIR.0000078635.89229.8A 12796124

[B50] Addya S Shiroto K Turoczi T Zhan L Kaga S Fukuda S Surrey S Duan LJ Fong GH Yamamoto F Maulik N Ischemic preconditioning mediated cardioprotection is disrupted in heterozygous Flt-1 (VEGFR-1) knockout mice J Mol Cell Cardiol 2005 38 345 351 10.1016/j.yjmcc.2004.11.033 15698841

[B51] Patel A van de Poll MC Greve JW Buurman WA Fearon KC McNally SJ Harrison EM Ross JA Garden OJ Dejong CH Wigmore SJ Early stress protein gene expression in a human model of ischemic preconditioning Transplantation 2004 78 1479 1487 10.1097/01.TP.0000144182.27897.1E 15599312

[B52] Mateo J Garcia-Lecea M Cadenas S Hernandez C Moncada S Regulation of hypoxia-inducible factor-1 by nitric oxide through mitochondriadependent and -independent pathways Biochem J 2003 376 537 544 10.1042/BJ20031155 14531732 PMC1223794

[B53] Brüne B Zhou J Nitric oxide and superoxide: Interference with hypoxic signaling Cardiovasc Res 2007 75 275 282 10.1016/j.cardiores.2007.03.005 17412315

[B54] Li F Sonveaux P Rabbani ZN Liu S Yan B Huang Q Vujaskovic Z Dewhirst MW Li CY Regulation of HIF-1alpha stability through S-nitrosylation Mol Cell 2007 26 63 74 10.1016/j.molcel.2007.02.024 17434127 PMC2905600

[B55] Sumbayev VV Budde A Zhou J Brune B HIF-1 alpha protein as a target for S-nitrosation FEBS Lett 2003 535 106 112 10.1016/S0014-5793(02)03887-5 12560087

[B56] Brüne B Zhou J The role of nitric oxide (NO) in stability regulation of hypoxia inducible factor-1alpha (HIF-1alpha) Curr Med Chem 2003 10 845 855 10.2174/0929867033457746 12678687

[B57] Kimura H Weisz A Ogura T Hitomi Y Kurashima Y Hashimoto K D'Acquisto F Makuuchi M Esumi H Identification of hypoxia-inducible factor 1 ancillary sequence and its function in vascular endothelial growth factor gene induction by hypoxia and nitric oxide J Biol Chem 2001 276 2292 2298 11056166 10.1074/jbc.M008398200

[B58] Thomas DD Espey MG Ridnour LA Hofseth LJ Mancardi D Harris CC Wink DA Hypoxic inducible factor 1alpha, extracellular signalregulated kinase, and p53 are regulated by distinct threshold concentrations of nitric oxide Proc Natl Acad Sci USA 2004 101 8894 8899 10.1073/pnas.0400453101 15178764 PMC428443

[B59] Zhou J Fandrey J Schumann J Tiegs G Brüne B NO and TNF-alpha released from activated macrophages stabilize HIF-1alpha in resting tubular LLC-PK1 cells Am J Physiol Cell Physiol 2003 284 C439 446 12388069 10.1152/ajpcell.00294.2002

[B60] Fukumura D Kashiwagi S Jain RK The role of nitric oxide in tumour progression Nat Rev Cancer 2006 6 521 534 10.1038/nrc1910 16794635

[B61] Luciano JA Tan T Zhang Q Huang E Scholz P Weiss HR Hypoxia inducible factor-1 improves the actions of nitric oxide and natriuretic peptides after simulated ischemia-reperfusion Cell Physiol Biochem 2008 21 421 428 10.1159/000129634 18453749

[B62] Hausenloy DJ Yellon DM New directions for protecting the heart against ischaemia-reperfusion injury: targeting the reperfusion injury salvage kinase (RISK)-pathway Cardiovasc Res 2004 61 448 460 10.1016/j.cardiores.2003.09.024 14962476

[B63] Ndiaye M Chataigneau M Lobysheva I Chataigneau T Schini-Kerth VB Red wine polyphenol-induced, endothelium-dependent NO-mediated relaxation is due to the redox-sensitive PI3-kinase/Akt-dependent phosphorylation of endothelial NO-synthase in the isolated porcine coronary artery FASEB J 2005 19 455 457 15623569 10.1096/fj.04-2146fje

[B64] Gao X Zhang H Takahashi T Hsieh J Liao J Steinberg GK Zhao H The Akt signaling pathway contributes to postconditioning's protection against stroke; the protection is associated with the MAPK and PKC pathways J Neurochem 2008 105 943 55 10.1111/j.1471-4159.2008.05218.x 18182053 PMC2746404

[B65] Zhu M Feng J Lucchinetti E Fischer G Xu L Pedrazzini T Schaub MC Zaugg M Ischemic postconditioning protects remodeled myocardium via the PI3K-PKB/Akt reperfusion injury salvage kinase pathway Cardiovasc Res 2006 72 152 162 10.1016/j.cardiores.2006.06.027 16901477

[B66] Issekutz TB Effects of six different cytokines on lymphocyte adherence to microvascular endothelium and in-vivo lymphocyte migration in the rat J Immunol 1990 144 2140 2146 2107253

[B67] Meldrum DR Shenkar R Sheridan BC Cain BS Abraham E Harken AH Hemorrhage activates myocardial NFkB and increases tumor necrosis factor in the heart J Mol Cell Cardiol 1997 29 2849 2854 10.1006/jmcc.1997.0506 9344778

[B68] Yabe Y Kobayashi N Nishihashi T Takahashi R Nishikawa M Takakura Y Hashida M Prevention of neutrophil-mediated hepatic ischemia/reperfusion injury by superoxide dismutase and catalase derivatives J Pharmacol Exp Ther 2001 298 894 899 11504782

[B69] Lindemann S Sharafi M Spiecker M Buerke M Fisch A Grosser T Gierer C Ibe W Meyer J Darius H NO reduces PMN adhesion to human vascular endothelial cells due to downregulation of ICAM-1 mRNA and surface expression Thromb Res 2000 97 113 123 10.1016/S0049-3848(99)00162-0 10680642

[B70] Liu P Xu B Hock CE Nagele R Sun FF Wong PY NO modulates P-selectin and ICAM-1 mRNA expression and hemodynamic alterations in hepatic I/R Am J Physiol 1998 275 H2191 198 9843819 10.1152/ajpheart.1998.275.6.H2191

[B71] Shimamura T Zhu Y Zhang S Jin MB Ishizaki N Urakami A Totsuka E Kishida A Lee R Subbotin V Furukawa H Starzl TE Todo S Protective role of nitric oxide in ischemia and reperfusion injury of the liver J Am Coll Surg 1999 188 43 52 10.1016/S1072-7515(98)00259-2 9915241 PMC3018864

